# HPV-Positive and -Negative Cervical Cancers Are Immunologically Distinct

**DOI:** 10.3390/jcm11164825

**Published:** 2022-08-18

**Authors:** Andris M. Evans, Mikhail Salnikov, Steven F. Gameiro, Saman Maleki Vareki, Joe S. Mymryk

**Affiliations:** 1 Department of Microbiology and Immunology, The University of Western Ontario, London, ON N6A 3K7, Canada; 2 Department of Oncology, The University of Western Ontario, London, ON N6A 5W9, Canada; 3 Department of Pathology and Laboratory Medicine, The University of Western Ontario, London, ON N6A 3K7, Canada; 4 London Regional Cancer Program, Lawson Health Research Institute, London, ON N6A 5W9, Canada; 5 Department of Otolaryngology, The University of Western Ontario, London, ON N6A 5W9, Canada

**Keywords:** human papillomavirus, cervical cancer, TCGA, gene expression, immune landscape, immune exhaustion, T-cell function, tumor immunology, neoantigens, TCR repertoire

## Abstract

Although infection with human papillomavirus (HPV) is associated with nearly all cervical cancers (CC), a small proportion are HPV-negative. Recently, it has become clear that HPV-negative CC represent a distinct disease phenotype compared to HPV-positive disease and exhibit increased mortality. In addition, variations between different HPV types associated with CC have been linked to altered molecular pathology and prognosis. We compared the immune microenvironments of CC caused by HPV α9 species (HPV16-like), HPV α7 species (HPV18-like) and HPV-negative disease. HPV-negative CC appeared distinct from other subtypes, with greatly reduced levels of lymphocyte infiltration compared to either HPV α9 or α7 CC. Besides reduced levels of markers indicative of B, T, and NK lymphocytes, the expression of T-cell effector molecules, activation/exhaustion markers, and T-cell receptor diversity were also significantly lower in HPV-negative CC. Interestingly, HPV-negative CC expressed much higher levels of potential neoantigens than HPV-positive CC. These results identify profound differences between the immune landscape of HPV-positive and HPV-negative CC as well as modest differences between HPV α9 and α7 CC. These differences may contribute to altered patient outcomes between HPV-negative and HPV-positive CC and potentially between CC associated with different HPV types.

## 1. Introduction

Worldwide, cervical cancer (CC) is the fourth most prevalent cancer in women, with over 600,000 new cases and 340,000 deaths in 2020 [1]. CC remains a leading cause of cancer death in younger women in economically disadvantaged countries [1]. Infection with human papillomavirus (HPV) is associated with 85–90% of CC [2,3], with no clear etiology for HPV-negative CC [4]. HPVs are highly transmissible, representing the most common sexually transmitted infection in North America [5]. Of the over 400 known HPV types, 30 preferentially infect the anogenital mucosa, resulting in papillomas (warts) [6,7]. A subset of HPV-induced lesions of the cervix progresses to carcinoma, commonly as a result of damage to the viral genome, leading to random integration into the host genome. This often leads to a constitutively elevated level of expression of the viral E6 and E7 oncogenes in CC [7].

Of the various cancer-causing HPV types, HPV16 is the predominant species, accounting for nearly 60% of all CC [2,3]. HPV16 is a member of the α9 family, which includes the closely related types HPV31, 33, 35, 52, 58, and 67 [6]. Statistically, HPV18 and HPV45 exhibit the second and third highest association with CC, accounting for ~10% and ~5% of cases, respectively [2,3]. HPV18 and 45 are members of the α7 family, which includes closely related types HPV39, 59, 68, 70, 85, and 97 [6]. Patients with HPV-positive CC appear to have a more favorable prognosis than their HPV-negative counterparts [8,9,10,11], and it is clear that HPV-positive and HPV-negative diseases are clinically and pathologically distinct [12,13]. Although somewhat controversial, patient outcomes for HPV16-positive CC appear superior to those that are HPV18-positive [10,14,15,16]. From a tumor virus perspective, these results may not be surprising, given the divergence in sequence and molecular function between the oncogenes encoded by the different HPV types [17].

The aim of this study was to compare the tumor immune landscape between HPV α9, HPV α7, and HPV-negative CC, with the goal of identifying differences that have implications in the diagnosis, prognosis, and treatment of these cancers. Despite the differences in pathology and clinical outcomes between HPV-positive and HPV-negative CC, few studies have directly compared the tumor immune landscapes between these distinct cancers of the cervix [18,19]. Given the tremendous impact that T-cell-targeting immune checkpoint inhibitors have had on cancer treatment [20] and the recent approval of these drugs for some CC [21], we undertook a detailed T-cell-centric analysis of the tumor immune landscape differences between HPV α9 (HPV16-like), HPV α7 (HPV18-like), and HPV-negative CC. Significantly increased T-cell infiltration, T-cell receptor (TCR) repertoire diversity, effector gene expression, activation status, and exhaustion marker expression were observed in both α9 and α7 HPV-positive CC as compared to HPV-negative CC. These observations provide strong evidence that the immune landscape of HPV-negative CC exhibits a distinct immune-cold phenotype compared to the corresponding HPV-positive disease. Intriguingly, HPV-negative CCs express a larger array of potential neoantigens compared to HPV-positive CC, which typically increases the likelihood of T-cell recognition, and is often associated with improved clinical outcomes [22]. These differences in the immune landscape may contribute to increased mortality associated with HPV-negative CC and suggest that it may be less amenable to immunomodulatory interventions such as immune checkpoint inhibitors.

## 2. Materials and Methods

### 2.1. Sample Collection and Ethics

All data from The Cancer Genome Atlas (TCGA) were downloaded via the Broad Genome Data Analysis Center’s Firehose server (https://gdac.broadinstitute.org/, accessed on 2 March 2017) or other publicly available sources as noted below; therefore, no ethical approval was needed.

### 2.2. Analysis of Cellular mRNA Expression

Level 3 mRNA expression data for the TCGA CC dataset was sourced from Broad Genome Data Analysis Center’s Firehose server (https://gdac.broadinstitute.org/, accessed on 2 March 2017), with the datasets manually annotated for HPV status [18,23,24]. For samples infected with multiple HPV types, the genotype with the highest expression was selected [25]. The CC RNA-sequencing dataset is comprised of 278 HPV-positive, 19 HPV-negative, and 3 normal control tissues. Of these, there are 165 HPV16-, 40 HPV18-, 1 HPV30-, 6 HPV31-, 9 HPV33-, 6 HPV35-, 5 HPV39-, 22 HPV45-, 1 HPV51-, 8 HPV52-, 1 HPV56-, 6 HPV58-, 3 HPV59-, 2 HPV68-, 1 HPV69-, 1 HPV70-, and 1 HPV73-positive samples. The correlation of cellular gene mRNA expression and HPV status was performed via sorting the dataset into 200 HPV α9 (HPV16, 31, 33, 35, 52, and 58 types; 200 samples total), HPV α7 (HPV18, 39, 45, 59, 68, and 70 types; 73 samples total) and HPV-negative CC (19 samples total) or normal (non-cancerous; 3 samples total) subsets, with subsequent calculations performed with R’s built-in wilcox.test function with the conf.level parameter set to 0.95. *q*-Values were calculated for each comparison group with a false discovery rate (FDR) of 10%.

### 2.3. Analysis of Immune Landscape Features

Selected immune landscape features for the TCGA CC datasets were extracted from Thorsson et al. [26] and similarly analyzed as above via sorting the samples into HPV α9, HPV α7, and HPV-negative CC subsets. As not all data necessary for the calculation of each immune landscape feature were available for all individual TCGA samples, these comparisons include only 196 HPV α9, 72 HPV α7 and 18 HPV-negative CC. HPV integration status was extracted from Qiu et al. [18] and used to compare immune landscape features between CC samples with integrated vs. non-integrated HPV genomes. This work identified 163 integrated and 29 non-integrated HPV α9 CC and 67 integrated and 1 non-integrated HPV α7 CC. Immune landscape comparisons between CC and head and neck cancers utilized TCGA level 3 mRNA expression data sourced from Broad Genome Data Analysis Center’s Firehose server (https://gdac.broadinstitute.org/, accessed on 2 March 2017), that was manually annotated for HPV status as described [27].

### 2.4. Survival Analysis

Survival analyses utilized the TCGA overall survival (OS) data from Liu et al. [28]. The correlation of survival and cellular gene mRNA expression was performed via sorting the dataset into HPV α9 and α7 subsets. Patients were dichotomized by median expression of CD96, CTLA4, LAG3, PD1, TIGIT, TIM3, CD39, or CD73, with subsequent calculations performed via the coxph and Surv functions, both available via the R survival packages.

## 3. Results

### 3.1. HPV-Positive and HPV-Negative CC Exhibit Strong Differences in Lymphocyte Infiltration

**Figure 1:**
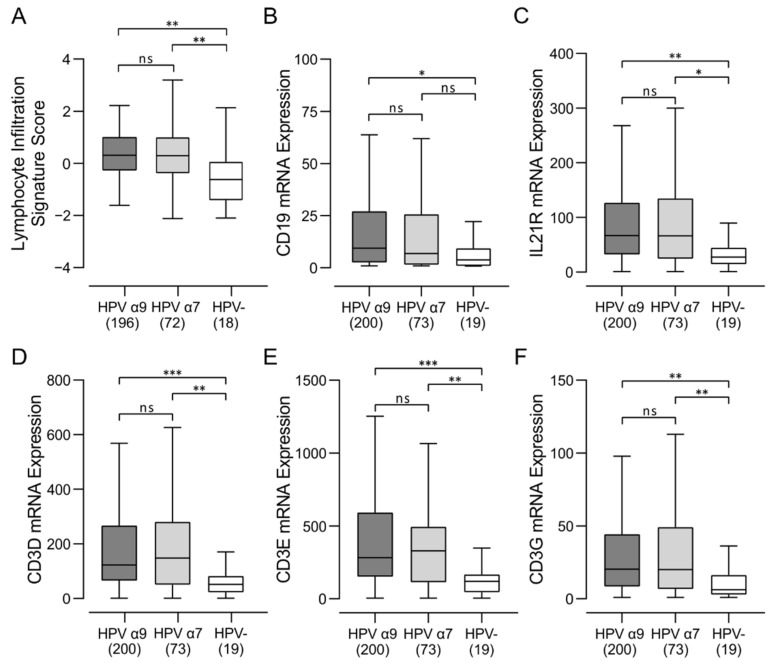
Analysis of tumor infiltrating lymphocytes and subsets in HPV-positive (HPV α9 and HPV α7) and HPV-negative (HPV-) cervical cancer. (**A**) Comparison of Lymphocyte Infiltration Signature Score between HPV α9, HPV α7, and HPV-negative cervical cancers. Values were extracted from Thorsson et al. [26] and HPV status annotated manually; (**B**–**F**) expression of marker genes related to B-cell (CD19, IL21R) and T-cell (CD3D, CD3E, CD3G) infiltration. Numbers in brackets refer to the number of samples included in each analysis. *** *p* ≤ 0.001, ** *p* ≤ 0.01, * *p* ≤ 0.05, ns (not significant).

Recent breakthroughs in cancer immunotherapy have clearly demonstrated the critical role of various immune mechanisms in controlling many different types of malignancies. The number, localization, and phenotypes of tumor-infiltrating lymphocytes (TILs) provides insight into the tumor immune landscape and may help predict response to immunotherapy [29]. We first started to explore the differences in the immune landscape between HPV-positive and HPV-negative CC by analyzing the TCGA Illumina HiSeq mRNA expression data from the CC cohort using a previously described Lymphocyte Infiltration Signature Score [26]. Samples from this cohort were divided into HPV α9 (HPV16-like; 196 samples), HPV α7 (HPV18-like; 72 samples) and HPV-negative CC (18 samples). Both groups of HPV-positive samples exhibited dramatically greater scores for lymphocyte infiltration compared to HPV-negative samples, indicative of substantial differences in the presence of TILs between HPV-positive CC compared to HPV-negative CC regardless of HPV type (Figure 1A). In this respect, HPV-negative CC appear to represent an immune-cold microenvironment, with characteristics of an immune-excluded tumor [30]. However, this mRNA based signature provides no spatial context for the localization of the lymphocytes within and around the tumor, which plays a critical role in immune effects against the tumor [29].

### 3.2. Higher Levels of B, T, and NK Lymphocytes Are Present in HPV-Positive CC

**Figure 2:**
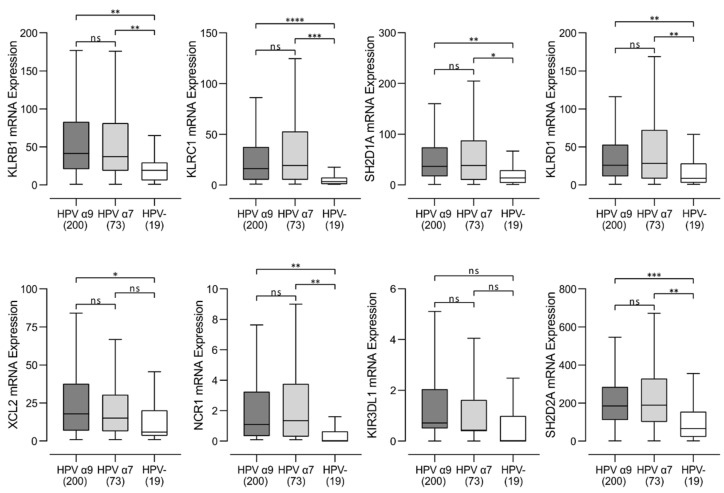
Transcript levels of NK cell marker genes in HPV-positive (HPV α9 and HPV α7) and HPV-negative (HPV-) cervical cancer. Normalized RNA-seq data was extracted from The Cancer Genome Atlas (TCGA) database for cervical cancer cohort. Numbers in brackets refer to the number of samples included in each analysis. **** *p* ≤ 0.0001, *** *p* ≤ 0.001, ** *p* ≤ 0.01, * *p* = 0.05, ns (not significant).

We next assessed the relative proportions of B, T, and NK lymphocytes in these CC samples, based on relative expression of mRNAs encoding lineage defining marker genes. We used the levels of CD19 and IL21R mRNA as measures of B-cell infiltration and CD3D, CD3E, and CD3G for T-cell infiltration and numerous markers for NK cells (Figure 1B–F and Figure 2). Values for normalized mRNA expression levels were available for 200 HPV α9, 73 HPV α7, and 19 HPV-negative CC. Higher levels of these markers of B, T, and NK cells were typically observed in HPV-positive CC compared to HPV-negative CC, regardless of HPV type.

### 3.3. Higher Levels of CD4^+^, CD8^+^ T Cells, and T Regulatory Cells Were Present in HPV-Positive CC

**Figure 3:**
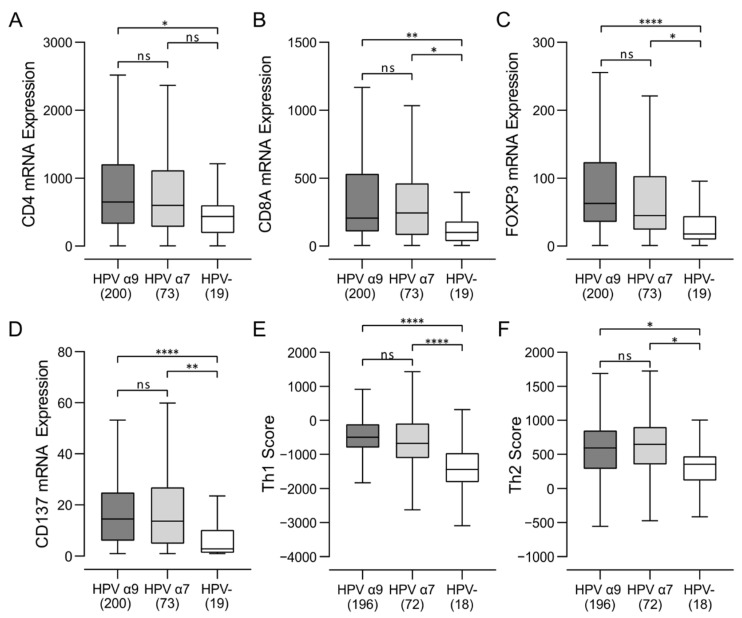
Analysis of T-cell populations in HPV-positive and HPV-negative cervical cancers. (**A**–**C**) Expression of marker genes related to CD4^+^ helper (CD4), CD8^+^ cytotoxic (CD8A), and Treg (FOXP3) cells; (**D**) expression of CD137 (TNFRSF9/4-1BB), an activation-induced costimulatory molecule present primarily on CD8^+^ T cells. (**E**,**F**) Comparison of CD4^+^ helper Th1 and Th2 Signature Score between HPV α9, HPV α7, and HPV-negative (HPV-) cervical cancers. Numbers in brackets refer to the number of samples included in each analysis. **** *p* ≤ 0.0001, ** *p* ≤ 0.01, * *p* ≤ 0.05, ns (not significant).

We next assessed the relative proportion of CD4^+^, CD8^+^ T cells and T regulatory cells (Tregs) in these samples, based on relative expression of the lineage defining CD4, CD8A, and FOXP3 marker genes, respectively (Figure 3). HPV-positive samples showed significantly increased expression of all three genes versus HPV-negative CC, confirming that enhanced infiltration by CD4^+^ T helper cells, CD8^+^ cytotoxic T cells, and Tregs are a common feature of HPV-positive CC as compared to HPV-negative samples. CD4^+^ T helper signatures suggest that all CC is biased towards a humoral Th2, rather than cell-mediated Th1 response, although both are upregulated in HPV-positive samples compared to HPV-negative CC. Furthermore, CD137 (4-1BB), an activation-induced costimulatory molecule present primarily on CD8^+^ T cells, was expressed at significantly higher levels in HPV-positive CC as compared to HPV-negative CC, suggesting a higher overall level of T-cell activation in HPV-positive CC (Figure 3).

### 3.4. HPV-Positive CC Expressed Higher Levels of Multiple T-Cell Effector Molecules Than HPV-Negative CC with Characteristics of a T-Cell-Inflamed Phenotype

**Figure 4:**
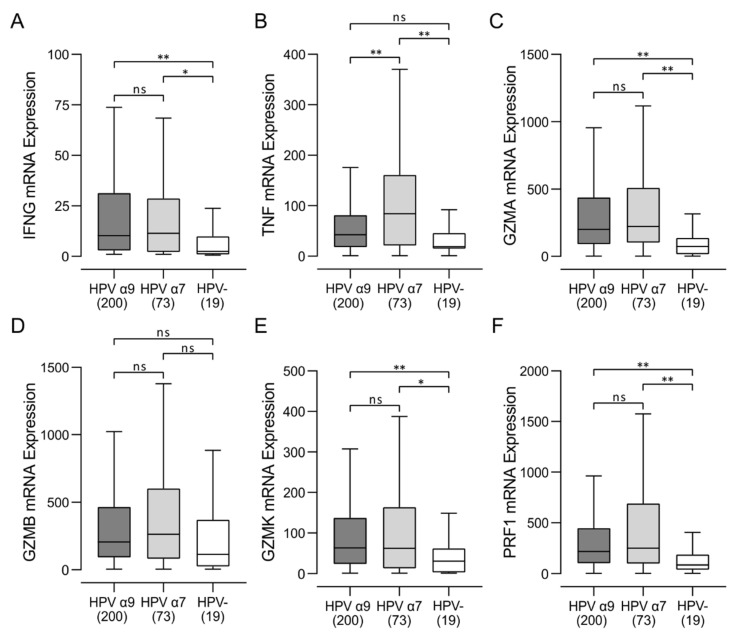
Transcript levels of lymphocyte effector molecules in HPV-positive (HPV α9 and HPV α7) and HPV-negative (HPV-) cervical cancers. (**A**–**F**) Expression of marker genes related to activated cytotoxic CD8^+^ T cells, including IFN-γ (IFNG), TNF-α (TNF), granzyme A (GZMA), granzyme B (GZMB), granzyme K (GZMK), and perforin (PRF1). Numbers in brackets refer to the number of samples included in each analysis. ** *p* ≤ 0.01, * *p* ≤ 0.05, ns (not significant).

Activated cytotoxic CD8^+^ T cells produce various effector molecules, including IFN-γ and TNF. Although expression of IFN-γ and TNF mRNAs in these samples was low, it was detected at significantly higher levels in HPV-positive versus HPV-negative CC (Figure 4). HPV-positive CC tumors also expressed significantly higher levels of cytotoxic mediators, including granzyme A (GZMA), granzyme B (GZMB), granzyme K (GZMK), and perforin (PRF1) compared to HPV-negative CC (Figure 4). Taken together, these results indicate that CD8^+^ T cells are not only present at higher levels in HPV-positive CC but are concomitantly more active and actively produce effector molecules such as IFN-γ, TNF, granzymes, and perforin.

**Figure 5:**
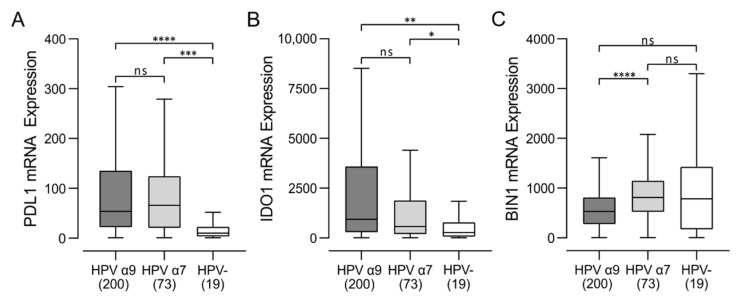
Transcript levels of tumor-derived interferon-responsive immunomodulatory genes in cervical cancer. Normalized RNA-seq data for genes associated with tumor cell mediated immunomodulation, including (**A**) PDL1 (CD274), (**B**) IDO1, and (**C**) its negative regulator BIN1, were compared between HPV-positive (HPV α9 and HPV α7) and HPV-negative (HPV-) cervical cancers. Numbers in brackets refer to the number of samples included in each analysis. **** *p* ≤ 0.0001, *** *p* ≤ 0.001, ** *p* ≤ 0.01, * *p* ≤ 0.05, ns (not significant).

Increased T-cell infiltration and higher levels of effector gene expression in HPV-positive CC indicates that these tumors exhibit many characteristics of T-cell-inflamed tumors [30]. Indeed, HPV-positive CC express high levels of the immune regulatory genes including PDL1 and IDO (Figure 5), another distinctive characteristic of the T-cell-inflamed tumor phenotype. We noted that expression of BIN1, a negative regulator of IDO1 expression [31], was significantly downregulated in HPV α9 CC, which express the highest levels of IDO1 (Figure 5). Overall, these data indicate that HPV-positive CC, in treatment-naïve patients, exhibits characteristics resembling a T-cell-inflamed phenotype compared to their HPV-negative counterparts (Figure 3 and Figure 4).

### 3.5. HPV-Positive CC Express High Levels of Multiple Immune Checkpoint Markers

**Figure 6:**
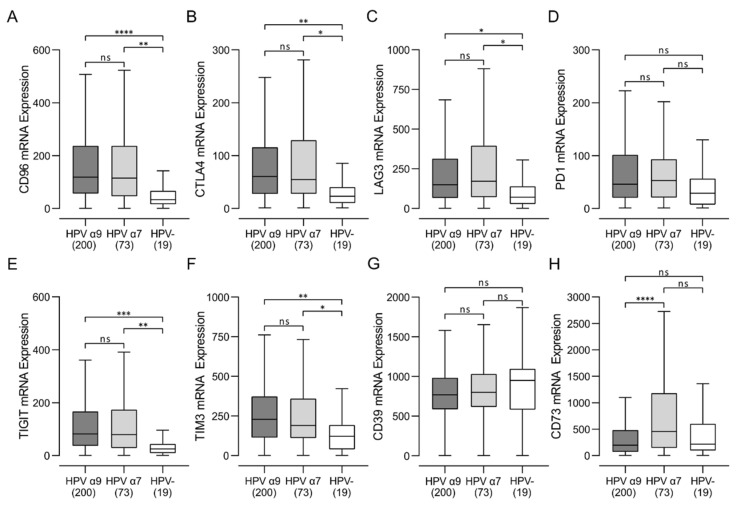
Analysis of immune checkpoint markers in HPV-positive (HPV α9 and HPV α7) and HPV-negative (HPV-) cervical cancers. (**A**–**F**) Expression of marker genes related to T-cell exhaustion markers, including CD96, CTLA4, LAG3, PD1 (PDCD1), TIGIT, and TIM3 (HAVCR2). (**G**,**H**) Expression of marker genes related to immunosuppressive purinergic signals including CD39 (ENTPD1) and CD73 (NT5E). Numbers in brackets refer to the number of samples included in each analysis. **** *p* ≤ 0.0001, *** *p* ≤ 0.001, ** *p* ≤ 0.01, * *p* ≤ 0.05, ns (not significant).

Once activated, T cells upregulate expression of multiple cell surface receptors that negatively regulate their proliferation and moderate their level of activation [32]. These immune checkpoint/exhaustion markers include CD96, CTLA4, LAG3, PD1, TIGIT, and TIM3, and a high expression level of these markers is another distinctive characteristic of T-cell-inflamed tumors [30]. These gene products are also important targets for immune checkpoint inhibitors that are approved, or under development. Notably, all these checkpoint genes, except for PD1, were significantly upregulated in HPV-positive CC compared with HPV-negative CC (Figure 6). Although a trend of increased PD1 expression was also present for HPV-positive versus HPV-negative CC, this was not statistically significant. Taken together, HPV-positive CC displays a markedly increased T-cell exhaustion signature from HPV-negative CC, indicative of sustained CD8^+^ T-cell activation reminiscent of a T-cell-inflamed phenotype. In contrast HPV-negative CC appear dramatically less immune-infiltrated, with a T-cell-excluded microenvironment.

We also examined the expression of the immunosuppressive molecule CD39 (ENTPD1) and its companion molecule CD73 (NT5E) in this cohort of patients. CD39 and CD73 encode enzymes that help calibrate the duration, magnitude, and chemical nature of purinergic signals delivered to immune cells. Together they control the shift from an ATP-driven proinflammatory environment to an adenosine-induced anti-inflammatory milieu [33]. Although their expression in Treg lymphocytes is induced by TCR activation [33], they can also be expressed by tumor cells and myeloid cells [34]. While CD73 showed significantly higher RNA expression levels in HPV α7-positive CC tumors compared to HPV α9 and HPV-negative CC samples, CD39 was not differentially expressed (Figure 6).

**Figure 7:**
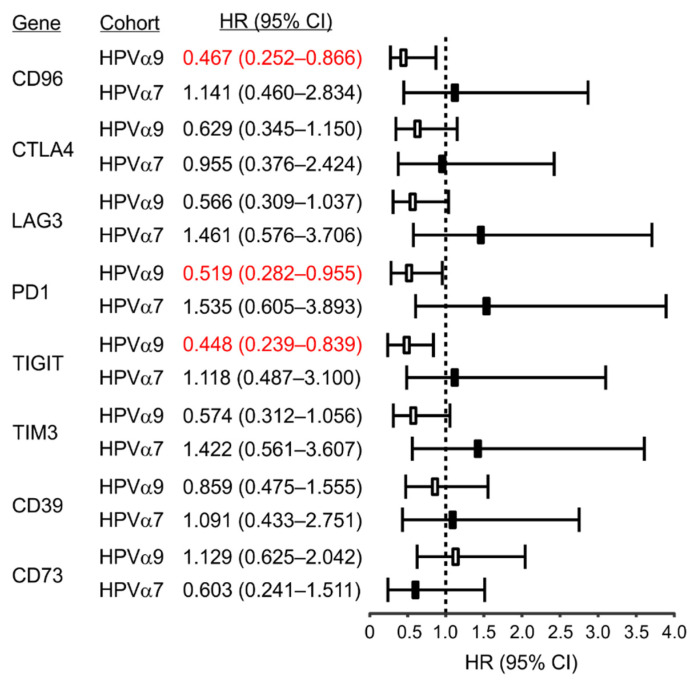
Correlation of immune checkpoint markers with patient mortality in HPV-positive (HPV α9 and HPV α7) cervical cancer. Hazard ratios (HR) and 95% confidence intervals (CI) related to immune checkpoint/T-cell exhaustion marker expression for CD96, CTLA4, LAG3, PD1 (PDCD1), TIGIT, TIM3 (HAVCR2), and genes related to immunosuppressive purinergic signals, including CD39 (ENTPD1) and CD73 (NT5E), were calculated. Numbers indicated in red are statistically significant.

Intriguingly, high expression of each of the five exhaustion factors, but not CD39 or CD73, was associated with a trend in reduced mortality in HPV α9 CC, which was significant for CD96, PD1, and TIGIT (Figure 7; see Figure S1). This correlation with survival further supports a critical role of T-cell activation in HPV α9 CC resolution. In contrast, no significant associations between exhaustion markers and survival were seen for HPV α7. While not significant, high expression of these markers trended towards increased mortality in HPV α7 CC (Figure 7). No correlations were performed for HPV-negative CC as the sample size was too small for statistical analysis.

### 3.6. Comparison of the T-Cell Receptor Repertoire between HPV-Positive and HPV-Negative CC

**Figure 8:**
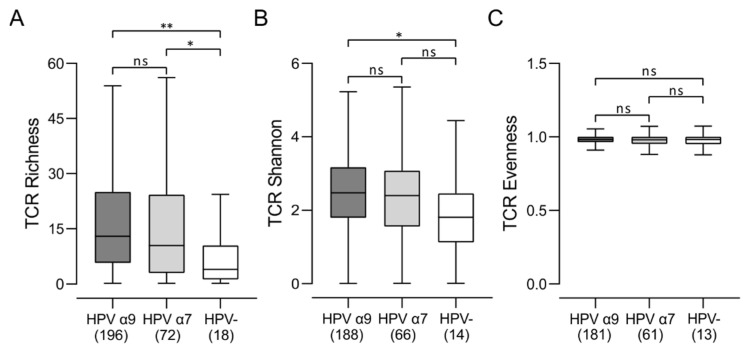
Comparison of the T-cell receptor (TCR) repertoire between HPV-positive (HPV α9 and HPV α7) and HPV-negative (HPV-) cervical cancers. (**A**) Comparison of unique TCR sequences in the TCR repertoire (richness); (**B**) comparison of clonal diversity weighted by the abundance of each complementarity-determining region 3 (Shannon entropy); (**C**) comparison of the distribution spectrum of TCR sequences, reflecting the relative abundance of individual T-cell clones (evenness). Numbers in brackets refer to the number of samples included in each analysis. ** *p* ≤ 0.01, * *p* ≤ 0.05, ns (not significant).

There is increasing evidence that analysis of TCR repertoire using deep sequencing approaches can serve as a biomarker of immune response in cancer patients [35]. We next compared the characteristics of the TCR repertoire between HPV-positive CC and HPV-negative CC (Figure 8). In terms of unique TCR sequences in the TCR repertoire (richness), both HPV α9 and HPV α7 CC groups showed an increased number of T-cell clones, as compared to HPV-negative CC. Measurements of clonal diversity weighted by the abundance of each complementarity-determining region 3 (CDR3; Shannon entropy) [36] revealed greater diversity in HPV α9 vs. HPV-negative CC, with a similar trend observed for HPV α7 CC. The distribution spectrum of these sequences, reflecting the relative abundance of individual T-cell clones (evenness), showed no significant differences between either group of HPV-positive CC or HPV-negative CC. These results indicate that the TCR repertoire in HPV-positive CC is significantly wider and more diverse than HPV-negative CC.

### 3.7. HPV-Negative CC Express High Levels of Potential Neoantigens

**Figure 9:**
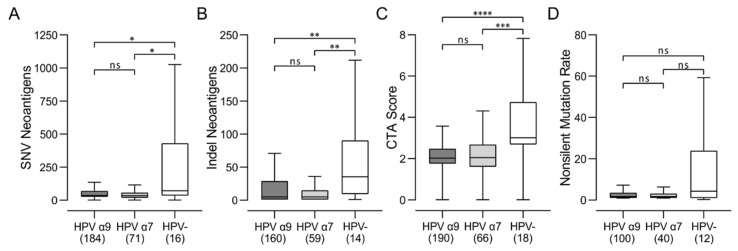
Comparison of the levels of potential neoantigens in HPV-positive (HPV α9 and HPV α7) and HPV-negative (HPV-) cervical cancers. Predicted levels of (**A**) single-nucleotide variant (SNV) neoantigens; (**B**) insertion–deletion (indel) neoantigens; (**C**) cancer testis antigen (CTA) score; and (**D**) non-silent mutations. Numbers in brackets refer to the number of samples included in each analysis. **** *p* ≤ 0.0001, *** *p* ≤ 0.001, ** *p* ≤ 0.01, * *p* ≤ 0.05, ns (not significant).

HPV-positive CC maintain expression of various viral proteins, particularly those encoded by the E6 and E7 oncogenes [7]. These viral proteins are recognized as foreign antigens by the human immune system, likely enhancing T-cell responses in HPV-positive CC [37]. Epitope spreading from one dominant viral antigen to another, or to cell-derived tumor-associated antigens, may enhance tumor cell clearance [38]. T-cell recognition of HPV-negative CC, which by definition do not express any foreign viral antigens, will depend on neoantigens derived from mutated cellular genes or aberrant expression of cancer testis antigens (CTAs), which are immunogenic, highly cancer-specific proteins normally only expressed in immune-privileged testis germ cells [39,40]. We compared the levels of potential neoantigens between HPV α9, HPV α7, and HPV-negative CC (Figure 9). HPV-negative CC are predicted to express higher levels of single-nucleotide variant (SNV) neoantigens, insertion–deletion (indel) neoantigens, and neoantigens related to CTA score compared to their HPV-positive counterparts. There was also a trend towards higher nonsilent mutation rates, although this was not significant (Figure 9D). Thus, HPV-negative CC exhibit a higher calculated level of neoantigens than HPV-positive CC and have at least the theoretical potential to be readily recognized by the adaptive immune system via these neoantigens.

### 3.8. Comparison of the Immune Landscape between HPV α9-Positive CC with Integrated or Non-Integrated Viral Genomes

While the HPV genome is maintained episomally in a normal infection, integration into the host cell genome is a frequent event in CC [7]. The impact of integration of the viral genome on CC prognosis has been suggested to be negatively correlated with patient outcomes [41], although recent data do not fully support this conclusion [42]. We directly compared all the immune related parameters from this study between HPV α9-positive CC with integrated viral genomes and HPV α9-positive CC with non-integrated viral genomes. No significant differences were observed (Table S2). A similar comparison could not be performed for HPV α7-positive CC as all but one of the 68 samples was integrated [18].

## 4. Discussion

Despite well-recognized differences between the pathology [12,13] and clinical outcomes [8,9,10,11] between HPV-positive and HPV-negative CC, few if any studies have directly compared the tumor immune microenvironment between these distinct cancers of the cervix. Cancer is a complex disease, and it has become increasingly clear that patient outcomes depend greatly on crosstalk between the tumor and its local immune microenvironment [43]. Given the tremendous impact that immune checkpoint inhibitors targeting T cells have had on cancer treatment and the recent approval of these drugs for PD-L1 positive CC [20], we undertook a detailed T-cell-centric analysis of the tumor immune landscape differences between HPV-positive and HPV-negative CC. As HPV-positive CC is commonly caused by several distinct HPV species, we also compared the tumor immune microenvironments between CC associated with HPV α9 (HPV16-like) and HPV α7 (HPV18-like), the most common HPV species involved in CC. These distinct HPV species exhibit a number of molecular differences [17] and have been associated with different patient outcomes [10,14,15,16]. Notably, immunological differences between HPV α9 and HPV α7 have also not been systematically characterized.

In this study, we report the detailed immune characterization of HPV-positive α9 and α7 CC compared to HPV-negative CC. We performed a mechanistic analysis of the tumor immune microenvironment in treatment-naïve CC using data from the TCGA cohort. However, examination of only the RNA expression levels limits the ability to accurately confirm expression levels on each cell type. We addressed this limitation by using generally accepted immune lineage-specific markers or signatures developed and published by others [26]. We found that both HPV-positive α9 and α7 CC tumors exhibited much higher lymphocyte infiltration than HPV-negative CC (Figure 1). This included higher levels of B, T, and NK lymphocytes (Figure 1 and Figure S1). Both HPV-positive α9 and α7 CC tumors exhibited increased levels of CD4^+^ helper T cells, CD8^+^ cytotoxic T cells, and Tregs, with characteristics of a predominant Th2 humoral skewed immune phenotype compared to HPV-negative CC (Figure 3). Interestingly, higher T-cell infiltration into HPV-positive tumors was accompanied with high CD137 (4-1BB) gene transcript levels, suggesting greater T-cell activation. Limited comparable data is available from other studies directly comparing these immune characteristics in CC, but higher lymphocyte infiltration is a well described phenomenon in all HPV-dependent cancers [44], and Tregs specific for HPV antigens have been recovered from CC previously [45]. Previous studies have reported that TILs recovered from CC exhibit prominent Th2 skewing, consistent with the data reported here [46,47].

In agreement with the high CD137 mRNA levels in HPV-positive CC samples, we observed higher T-cell effector production in these CC samples (Figure 4). Indeed, HPV-positive CC expressed significantly higher levels of IFN-γ, TNF, perforin, and granzymes A, B, and K compared to HPV-negative CC samples. Notably, no significant differences in the expression of any of these effectors were observed between HPV α9 and α7 CC. Notably, these values for HPV-positive CC are generally lower than in HPV-positive head and neck squamous cell carcinoma (HNSCC) [48], and a direct comparison of these values is provided in Table S1. Although the mRNA expression data lack spatial data within the tumor, these analyses allow for a preliminary analysis of the immune microenvironments of treatment-naïve CC. The identification of potential biomarkers and targets from this research will guide future endeavors to investigate the potential of these markers more thoroughly.

Given the normalization of TCGA RNA-sequencing data, our calculated values for these T-cell markers and effector genes in HPV-positive CC can be directly compared with those found in HPV-positive HNSCC and represent approximately 25–50% of those values (Table S1). Thus, while these two HPV-dependent cancers exhibit a number of similarities in terms of immune microenvironment, the magnitude of inflammation is generally lower in HPV-positive CC than HPV-positive HNSCC.

Based on these analyses, it became apparent that HPV-positive CC exhibit many characteristics of T-cell-inflamed tumors [30] although at a reduced level to their HPV-positive HNSCC counterparts (Table S1). Indeed, HPV-positive CC express high levels of the immune regulatory genes including IDO and PDL1, indicative of the T-cell-inflamed tumor phenotype (Figure 5). The presence of these immunosuppressive events is generally accompanied by increased expression of multiple T-cell immune checkpoint molecules in a T-cell-inflamed microenvironment, which are also present in HPV-positive, but not HPV-negative CC (Figure 6). Indeed, expression of CD96, CTLA4, LAG3, TIM3, PD1, and TIGIT are all substantially higher in HPV-positive CC, although significant differences were not always present, particularly for comparisons of HPV α7 CC vs. HPV-negative CC (Figure 6). This agrees well with other studies reporting high expression of CD96, PD1, CTLA4, LAG3, PD1, and TIM3 protein in CC in general [49,50,51,52]. Notably, high expression of each of these five exhaustion factors was associated with a trend in reduced mortality in HPV α9 CC, which was significant for CD96, PD1, and TIGIT (Figure 7). This is very similar to what is observed in HPV-positive HNSCC, which is primarily HPV α9 [48]. In contrast, none of the exhaustion markers were associated with reduced mortality when expressed at higher levels in HPV α7 CC. Indeed, they actually trended towards decreased survival, although this was not significant (Figure 7). These data suggest that T-cell exhaustion markers, particularly PD1 and TIGIT, may represent a useful biomarker of survival in HPV α9 CC. The trend toward the opposite clinical association of increased mortality with high exhaustion marker expression in HPV α7 CC requires further investigation in a larger cohort. However, these results suggest that the immune microenvironment is indeed different between HPV α9 and HPV α7 CC. This is also reflected in the significant increase in TNF (Figure 4) and CD73 expression in HPV α7 CC vs. HPV α9 CC (Figure 6). In contrast, no significant differences were observed between HPV α9 CC with integrated HPV versus non-integrated HPV (Table S2).

In terms of the diversity of the TCR repertoire, both HPV α7 and α9 CC exhibit a significantly wider and more diverse T-cell response than HPV-negative CC (Figure 8). HPV-negative CC also clearly show a larger array of potential neoantigens compared to HPV-positive CC, as determined by a number of independent measures, including non-silent mutations, single-nucleotide variant (SNV) neoantigens, insertion–deletion (indel) neoantigens, and CTA score (Figure 9). Thus, HPV-negative CC have at least the theoretical potential to be readily recognized by the adaptive immune system via these neoantigens yet exhibit a relatively small TCR repertoire and limited numbers of TILs. Mechanistically, increased TIL levels and activity can be associated with lower tumor clonal diversity. This may arise as a consequence of immunoediting, where selective pressure by the immune system depletes tumor cell populations expressing target neoantigens [53]. Therefore, the lower level of immune infiltration exhibited by HPV-negative CC, and the corresponding lack of immunoediting, could result in the observed larger array of potential neoantigens compared to HPV-positive CC. Alternatively, this could also be related to the observation that tumor-specific T-cell dysfunction can be driven by persistent antigen exposure [54]. These data provide further evidence that the immune landscape in HPV-negative CC is immunologically cold. As HPV-negative CC exhibits a lower level of lymphocyte infiltration compared to HPV-positive CC (Figure 1, Figure 2 and Figure S1), this could occur via the process of immune exclusion [55].

Taken together, this study provides clear evidence, for the first time, that the immune landscape of HPV-positive CC represents a distinct tumor immune microenvironment from HPV-negative CC, consistent with many characteristics of a T-cell-inflamed phenotype, although the relative levels of these markers are lower than observed in HPV-positive HNSCC (Table S1). Notably, we identified multiple mechanisms that negatively regulate the anti-tumor immune response that are significantly upregulated in the majority of HPV-positive CC cases, several of which are correlated with improved clinical outcome in HPV α9 CC. These exhaustion markers may serve as useful biomarkers for survival in HPV α9 CC, as well as targets for immunotherapies [56]. Many of these negative regulators of the immune response are under investigation in current clinical trials and HPV-positive CC has many immunological features suggesting that it would be amenable to immune checkpoint inhibition therapy.

We also identified differences in NK-cell infiltration and NK-cell-based anti-cancer therapies are emerging as potentially useful for cancer treatment [57]. Like T cells, NK cells can be engineered to express chimeric antigen receptors (CARs) directed against antigens expressed on the surface of tumor cells [58]. These CAR NK cells can be safe and effective [59], and an initial trial using a TCR directed against HPV16 E6 showed some efficacy [60]. Other therapies target NK-cell inhibitory receptors or pathways. For example, monalizumab, an anti-KLRC1 blocking antibody, has been tested in several clinical trials, either as a single agent or in combination with other therapies [61]. The significantly higher expression of KLRC1 in HPV-positive CC compared to HPV-negative CC indicates that this therapy may be a beneficial treatment in HPV-positive CC.

In addition to the changes described above, subtle differences in the immune landscapes between HPV α9 and α7 CC suggest that these cancers may not respond identically to immune checkpoint inhibition. This is potentially important, as a recent large trial of the PD1 monoclonal antibody pembrolizumab did not consider HPV status or HPV type as factors that may contribute to clinical response [62]. Given that pembrolizumab is only approved for treatment of PDL1-positive CC [21], our data suggest that it is likely being used mainly for HPV-positive CC, as HPV-negative CC appear to express much lower levels of PDL1. Ultimately, immune-predictive biomarkers may pave the way for patient stratification for immunotherapy-based treatments currently in progress [63] or promising future combination therapies against multiple T-cell checkpoints [50].

## Data Availability

Not applicable.

## Supplementary Materials

The following supporting information can be downloaded at: https://www.mdpi.com/article/10.3390/jcm11164825/s1, Figure S1: Impact of expression of selected immune checkpoint markers on patient overall survival. Table S1: Comparison of immune marker scores between cervical cancer and head and neck cancer. Table S2: Comparison of immune marker scores between cervical cancer with integrated or non-integrated HPV α9.
