# Supplementary material for: HPV-Positive and -Negative Cervical Cancers Are Immunologically Distinct
Source: J Clin Med. 2022 Aug 18;11(16):4825. doi: 10.3390/jcm11164825 (PMC9410291; doi:10.3390/jcm11164825)
Supplement: Supplementary file 1 [file jcm-11-04825-s001.zip › Figure S1.pdf]

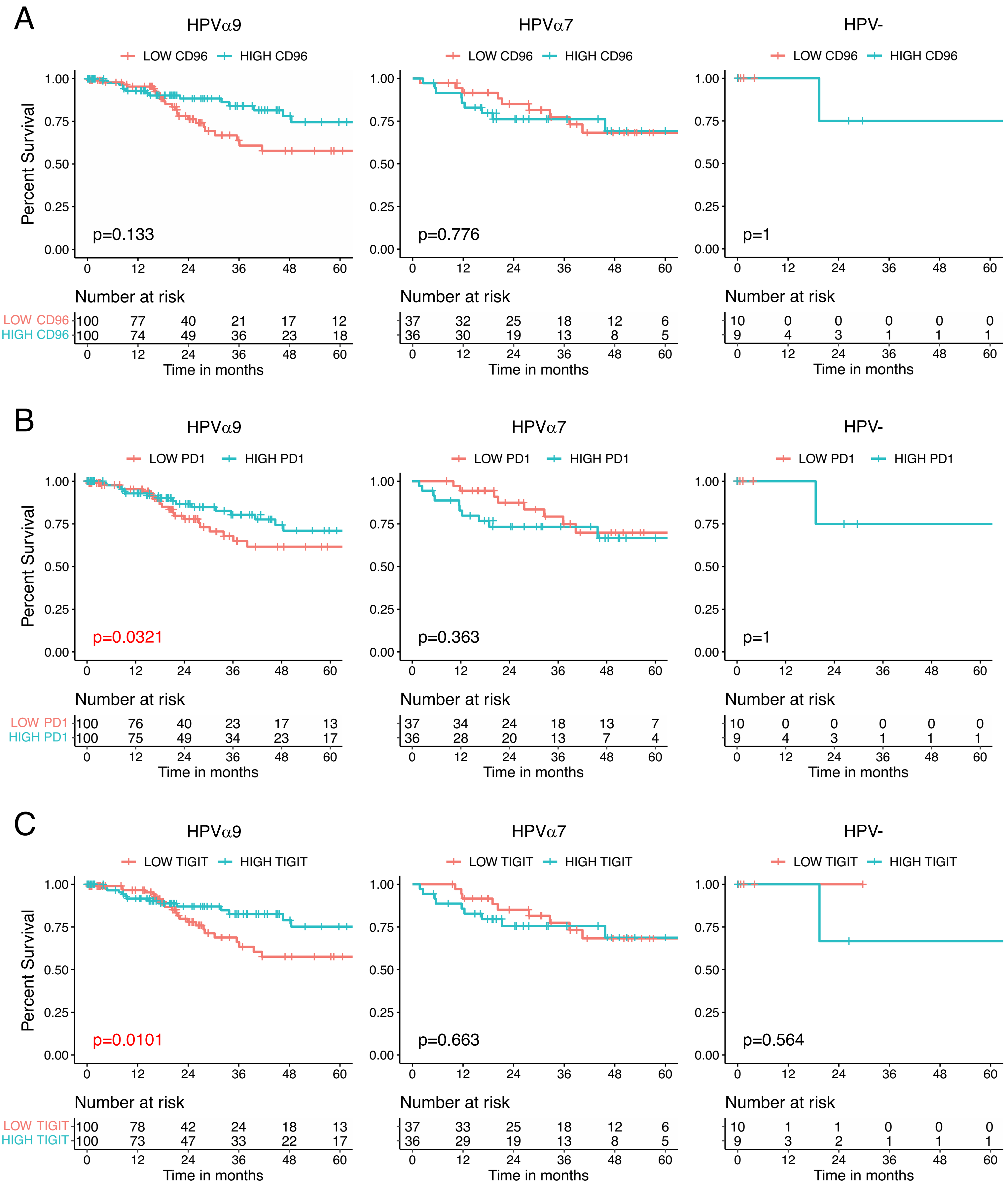

Figure S1: Impact of expression of selected immune checkpoint markers on patient overall survival. Kaplan-Meier analysis of patient survival for HPVα9, HPVα7 and HPV- patients dichotomized by median expression of CD96 (A), PD1 (B) or TIGIT (C). Statistically significant p values are indicated in red ($p<0.05$).
